# Unveiling the Pathogenic Bacteria Causing Descending Necrotizing Mediastinitis

**DOI:** 10.3389/fcimb.2022.873161

**Published:** 2022-06-08

**Authors:** Qiang Sun, Zixuan Li, Panpan Wang, Junfang Zhao, Shuai Chen, Minglei Sun

**Affiliations:** Department of Oral and Maxillofacial Surgery, The First Affiliated Hospital of Zhengzhou University, Zhengzhou, China

**Keywords:** maxillofacial infection, mediastinal infection, mNGS, pathogenic bacteria, early diagnosis and treatment

## Abstract

The combination of maxillofacial infections (MI) with descending necrotizing mediastinitis (DNM) is a complex disease characterized by rapid development and high mortality. Here, we performed metagenomic next-generation sequencing (mNGS) using samples from 21 patients with MI and eight patients with DNM. In this study, we found that the species richness of the DNM group was higher than that of the MI group, and the species diversity of the DNM group was higher than that of the MI group, with no statistically significant differences between groups (P > 0.05). LefSE analysis revealed that the main species differing between groups were *Bacillus*, *Lactobacillus*, *Streptococcaceae*, and *Streptococcus* (*S. constellatus* and *S. anginosus*). In addition, the PLS-DA analysis revealed that the dominant groups in the DNM group at the species level were *S. constellatus*, *S. anginosus*, *Streptococcus intermedius*, *Prevotella oris*, *Mogibacterium timidum*, and *Eubacterium nodatum*. Next, we correlated the clinical characteristics of the patients with the relative abundance of the pathogens identified in the LefSe and PLS-DA analyses. The relative abundance of *S. anginosus* was positively correlated with C-reactive protein (CRP) and calcitoninogen (PCT) but negatively correlated with the percentage of lymphocytes (Lymph%) (P < 0.05). On the other hand, *M. timidum* was positively correlated with the percentage of neutrophils (Neut%) and glycated hemoglobin (GLU) (P < 0.05), and *Parvimonas micra* was positively correlated with CRP (P < 0.05).

## Introduction

Oral and maxillofacial infections are caused by specific pathogenic bacteria or dysbiosis in the maxillofacial area. These infections can spread to surrounding tissues and interstitial spaces through weak resistance structures, causing severe complications such as cavernous sinus thrombophlebitis, brain abscess, sepsis, and mediastinitis along nerves and blood vessels (Reynolds and Chow, 2007; Bagheri, 2008). The spread of the infection to the mediastinum is caused by the pressure of the pus cavity, gravity, and negative intrathoracic pressure (Foroulis and Sileli, 2011), resulting in descending necrotizing mediastinitis (DNM). DNM is the most common and serious complication of oral and maxillofacial space infections, with a mortality rate of 61.5%, as it leads to sepsis and organfailure if not treated promptly (Freeman et al., 2000; Misthos et al., 2007; Kocher et al., 2012; Huang et al., 2015). Obtaining live pathogens, such as bacteria, fungi, and viruses, is the gold standard for diagnosing infectious diseases. However, *in vitro* culture of pathogens is generally time-consuming and cumbersome in terms of operational steps, and most pathogens cannot be cultured (Buehler et al., 2016; James et al., 2018). According to statistics, about 70% of patients with infectious diseases cannot receive timely and effective treatment because traditional testing methods are unable to identify the pathogen, thus worsening their condition (Morens and Fauci, 2012). Therefore, rapid, specific, and high-throughput pathogen detection methods are essential for effective diagnosis and timely prevention and treatment of infectious diseases. Metagenomic next-generation sequencing (mNGS) is fast, sensitive, and accurate (Liu et al., 2013; Simner et al., 2018). Hence, mNGS could be used as a new detection method for early clinical diagnosis and treatment of maxillofacial space infections (Ambardar et al., 2016; Gu et al., 2019).

Previously, Chen et al. compared and analyzed the differences between mNGS and conventional bacterial culture techniques to detect pathogenic bacteria in maxillofacial space infections and found that the average detection time was 18.81 ± 3.73 h for mNGS and 83.25 ± 11.64 h for bacterial culture. In addition, the positive detection rate was 100% for mNGS and 31.25% for conventional bacterial culture (Chen et al., 2021). Long et al. also performed mNGS and blood culture on plasma from 78 ICU patients, and the results showed a detection rate of 30.77% by mNGS, while the detection rate by blood culture was only 12.82%, indicating that mNGS has higher sensitivity in pathogen detection (Long et al., 2016). Li et al. tested lung tissue samples from 20 patients with suspected lung infections and showed that the sensitivity and specificity of bacterial identification by mNGS and the culture method were 100% and 76.5%, respectively, with a negative predictive value of 100% (Li et al., 2018).

Hence, mNGS can detect pathogens more rapidly and accurately than conventional tests. This allows for timely adjustment of antibiotics based on test results which can significantly reduce the morbidity and mortality rate of DNM. This study detects the differential microbiota of DNM due to maxillofacial space infections using mNGS and provides an experimental basis for early evidence-based medical diagnosis treatment and prognosis of maxillofacial space infections.

## Materials and Methods

### Sample Collection

This study collected pus specimens from 29 patients with maxillofacial space infections. The patients were divided into two groups according to the enhanced CT imaging of the maxillofacial, neck, and chest at the time of admission. One group of eight patients developed descending necrotizing mediastinitis (DNM group); the other group had a regular maxillofacial space infection (MI group). Samples from both groups were collected fresh during surgery. A 2 ml pus sample was extracted with a sterile syringe and stored at -80°C after being transferred to the laboratory with liquid nitrogen within half an hour. All patients and their families signed an informed consent form, and this study was approved by the Ethics Committee of the First Affiliated Hospital of Zhengzhou University (approval number: KY-2019-LW007).

### DNA Extraction From Pus Samples

Sample DNA extraction was performed using an MP’s Fast DNA Spin Kit following the indications below. The kit is suitable for the genome of all microorganisms in pus samples.

1) Add the 2 ml pus to the Lysing Matrix E tube.
2) Add 978 µl of the SPB and 122 µl of MT Buffer.
3) Vortex the tube for 40 to 50 s. Shake it from all angles to ensure homogeneous mixing. Then, centrifuge at 8000 r/min for 15 min.
4) After centrifugation, transfer the supernatant to a microcentrifuge tube, mix by adding 250 µl of PPS reagent, and shake the tube 10 times.
5) Centrifuge at 8000 r/min for 5 min and transfer the supernatant to a clean 5 ml centrifuge tube.
6) Vigorously shake the binding matrix and then aspirate 1 ml into the centrifuge tube in the previous step.
7) Shake the centrifuge tube up and down for 2 min. Wait 3 min for the silica matrix to precipitate.
8) Carefully remove 600 µl of supernatant and avoid touching the precipitated binding matrix. Discard the supernatant, transfer about 600 µl of the mixture to the SPIN filter, and centrifuge at 8000 r/min for 1 min.
9) Pour the liquid into the collection tube and repeat the previous step until the liquid in the 5 ml centrifuge tube is aspirated.
10) Add 500 µl of SEWS-M solution and mix well with a small gun. Centrifuge at 8000 r/min for 1 min, and then remove the liquid from the collection tube.
11) Repeat the previous step replacing the centrifuge tube with a 1.5 ml tube.
12) Centrifuge at 8000 r/min for 2 min to remove residual SEWS-M solution. Then replace with a clean 1.5 ml centrifuge tube.
13) Air dry the SPIN Filter for 5 min at room temperature.
14) Add 80 µL of DES solution to the SPIN filter. Carefully mix it with Binding Matrix using a small gun tip, and water bath at 65°C for 15 min. Centrifuge at 8000 r/min for 2 min to obtain about 50 µL of DNA solution. Add another 80 µL of DES solution and repeat the previous steps to obtain approximately 100 µl of DNA solution.
15) Discard the SPIN Filter and store the extracted DNA at -20°C for subsequent experiments.

### Library Preparation

The extracted DNA was fragmented using a Covaris M220 ultrasonicator, and fragments of approximately 300 bp were screened. Using the Tru SeqTM DNA Sample Prep Kit, a Y-shaped junction was attached to both ends of the fragmented DNA, the junction was removed by screening with magnetic beads, and the DNA was denatured using sodium hydroxide after PCR amplification to produce a single-stranded DNA fragment.

### Illumina Hiseq Sequencing

1) Add a modified DNA polymerase and dNTP with four fluorescent labels, synthesizing only one base per cycle.
2) Scan the surface of the reaction plate with a laser and read the nucleotides polymerized up by the first reaction of each template sequence.
3) Chemically cleave the “fluorescent group” and “termination group” to restore the 3’ end adhesion and continue the polymerization of the second nucleotide.
4) Count the results of the fluorescence signal collected in each round to obtain the sequence of the template DNA fragment.

### Data Quality Control

Using Seqprep (https://github.com/jstjohn/SeqPrep), the reads 3’ end and 5’ end adapter sequences for quality clipping. Reads with less than 50 bp in length, with average base mass values below 30, and those containing N bases were removed after clipping using Sickle (https://github.com/najoshi/sickle), retaining high-quality pair-end reads and single-end reads. The reads were then matched to the host DNA sequences using the BWA software (http://bio-bwa.sourceforge.net), and the contaminated reads with high similarity were removed. The removed host DNA sequences included those from GRC38.p13 and the Inflammation One reference genome

### Species Identification

Species identification was performed using kraken2/bracken (Garcia et al., 2021) and NT was chosen for the microbial database. The relative abundance of each species was calculated for each sample as “reads of that species/total microbial reads of that sample”. The species abundance in each sample was also counted at each taxonomic level of domain, kingdom, phylum, class, order, family, genus, and species to construct abundance profiles at the corresponding taxonomic levels.

### Experimental Data Analysis

Alpha diversity analyzes species diversity in a single sample and includes the Chao1 and Shannon indices (Jiang et al., 2015). The Chao1 index reflects the number of species in the sample microbiota (richness). In contrast, the Shannon index combines the number of species in the microbiota (richness) and the relative abundance of each species in the sample (evenness), expressed as species diversity in the microbiota.

Linear discriminant analysis effect size (LEfSe) (Fazlollahi et al., 2018) can be used to discover new biomarkers to identify the groups that best characterize each study group, and LDA analysis is primarily intended to find species that differ significantly in relative abundance between groups. LEfSe first detects differences in species abundance between groups using the non-parametric Kruskal-Wallis test to obtain species with significant differences; then, the Wilcoxon test was used to test the consistency of the differences between subgroups of the different groups in the first step, and finally, the LDA discriminant scores were used to estimate the magnitude of the effect of these differential species on intergroup distinctions. We considered taxa with LDA > 4 to be significant.

Sparse partial least squares discriminant analysis (sPLS-DA) (Hadj-Henni et al., 2021) aims to identify the key colonies that best distinguish between the two groups of samples, using the mixOmics package (Rohart et al., 2017) in the R language program for statistical analysis and graphing.

### Statistical Methods

Age, white blood cell (WBC) count, platelet count, neutrophil percentage, lymphocyte percentage, monocyte percentage, C-reactive protein, calcitoninogen, and glycosylated hemoglobin were tested by the Wilcoxon non-parametric rank-sum test in both groups. Gender, history of smoking, history of alcohol consumption, and history of diabetes mellitus were tested by chi-square test in both groups. Between-group diversity analysis and analysis of variance Wilcoxon non-parametric rank-sum test was used, and the relationship between phenotypic and clinical data and microbial communities was explored by Spearman correlation analysis. The test level p = 0.05.

## Results

### Clinical Information of the Samples

In this study, pus specimens were collected from 29 patients with maxillofacial space infections. Of these, eight were caused by descending necrotizing mediastinitis (DNM group) based on imaging, and 21 patients had a simple maxillofacial space infection (MI group). Among the 29 patients, 19 were male and 10 were female; the mean age was 54.48 ± 15.62 years; seven had a history of previous alcohol consumption, 11 had a history of smoking, and 14 had a history of previous diabetes mellitus (see **Table 1** for details of clinical data). Platelet count, neutrophil percentage, lymphocyte percentage, monocyte percentage, C-reactive protein (CRP), calcitoninogen (PCT), and glycated hemoglobin were compared in the two groups using a Wilcoxon non-parametric rank-sum test, and the results did not show statistically significant differences between the groups (P > 0.05). The chi-square test was used for gender, history of smoking, history of alcohol consumption, and history of diabetes in both groups, and the results did not show statistically significant differences between the two groups (P > 0.05).

**Table 1:** Clinical information of the two sample groups.

Factors	MI (n = 21)	DNM (n = 8)	P-value
**Gender**			0.27
Male	12	7	
Female	9	1	
**Age**	56.67 ± 16.30	48.75 ± 11.89	0.28
**Smoking**			0.21
Yes	6	5	
No	15	3	
**Drinking**			0.13
Yes	3	4	
No	18	4	
**Diabetes**			0.79
Yes	10	4	
No	11	4	
**WBC**	16.45 ± 5.16	17.20 ± 7.22	0.94
**PLT**	227.53 ± 111.04	182.00 ± 85.86	0.46
**Neut%**	85.61 ± 8.74	88.13 ± 7.54	0.55
**Lymph%**	6.82 ± 4.88	4.23 ± 2.66	0.18
**Mono%**	5.52 ± 2.60	6.85 ± 7.35	0.49
**CRP**	173.74 ± 103.74	212.01 ± 86.75	0.46
**PCT**	8.89 ± 20.99	10.32 ± 12.43	0.15
**GLU**	8.65 ± 5.89	11.06 ± 8.84	0.39

WBC, White blood cell; PLT, blood platelet count; Neut%, percentage of neutrophilic granulocyte; Lymph%, Percentage of lymphocytes; Mono%, Percentage of Monocyte; CRP, C-reactive protein; PCT, procalcitonin; GLU, Glycated hemoglobin.

### Data Quality Control and Cleaning Results

**Figure 1:**
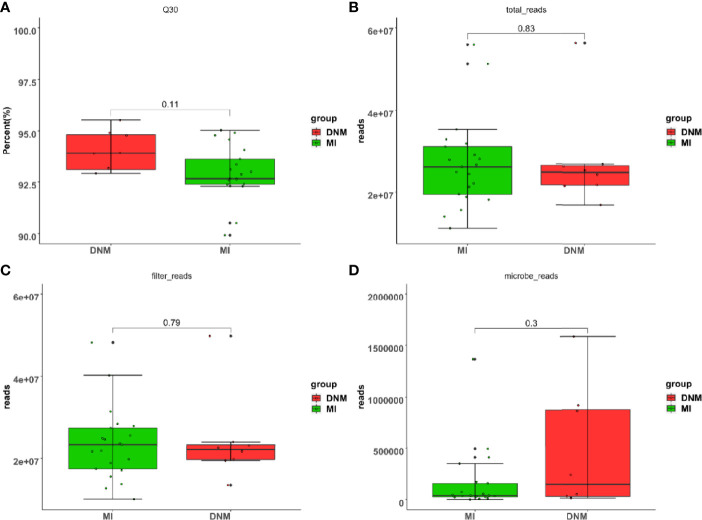
Data quality control sketch. **(A)** raw data Q30 scale statistics, **(B)** total reads, **(C)** filter reads, **(D)** microbe reads.

Next, the 29 pus samples were investigated using mNGS. The sequencing data were first subjected to data quality control and cleaning to ensure high data quality for the subsequent analysis steps (**Figure 1**). Data quality control included raw data Q30 statistics, the percentage of bases with base quality values greater than or equal to 30 on the Illumina sequencing platform. Total reads: the total number of nucleic acid sequences obtained by mNGS; filter reads, microbe reads: the total number of nucleic acid sequences of microorganisms detected in the sample after removing the human-derived nucleic acid sequences. A Wilcoxon non-parametric rank-sum test was performed between the two groups and showed no statistically significant difference (P > 0.05).

### mNGS Detection Results

A total of 131 pathogenic bacteria were detected in the DNM group, of which the top 10 in relative abundance were: *P. oris* (21.04%), *S. constellatus* (10.14%), *P. intermedia* (6.35%), *Prevotella buccae* (5.81%), *S. anginosus* (5.62%), *Peptostreptococcus stomatis* (5.02%), *P. micra* (4.67%), *Bacteroides heparinolyticus* (4.43%), *M. timidum* (4.23%), and *Streptococcus* sp. *NSJ-72* (4.15%) (**Table 2**).

**Table 2:** Top 10 species detected in DNM group.

Species	Relative abundance (%)	Gram stain	Tolerance to oxygen
*Prevotella oris*	21.04	G-	Anaerobic
*Streptococcus constellatus*	10.14	G+	Aerobic
*Prevotella intermedia*	6.35	G-	Anaerobic
*Prevotella buccae*	5.81	G-	Anaerobic
*Streptococcus anginosus*	5.62	G+	Part-time anaerobic
*Peptostreptococcus stomatis*	5.02	G+	Anaerobic
*Parvimonas micra*	4.67	G+	Anaerobic
*Bacteroides heparinolyticus*	4.43	G-	Anaerobic
*Mogibacterium timidum*	4.23	G+	Anaerobic
*Streptococcus* sp. *NSJ-72*	4.15	G+	Aerobic

A total of 127 pathogenic bacteria were detected in the MI group, of which the top 10 in relative abundance were: *P. oris* (18.59%), *P. micra* (8.41%), *Prevotella baroniae* (6.36%), *S. constellatus* (5.67%), *P. stomatis* (5.02%), *Mycolicibacterium houstonense* (4.76%), *Porphyromonas endodontalis* (4.56%), *P. buccae* (4.52%), *P. intermedia* (4.03%), and *B. heparinolyticus* (3.12%) (**Table 3**).

**Table 3:** Top 10 species detected in MI group.

Species	Relative abundance (%)	Gram stain	Tolerance to oxygen
*Prevotella oris*	18.59	G-	Anaerobic
*Parvimonas micra*	8.41	G+	Anaerobic
*Prevotella baroniae*	6.36	G-	Anaerobic
*Streptococcus constellatus*	5.67	G+	Aerobic
*Peptostreptococcus stomatis*	5.02	G+	Anaerobic
*Mycolicibacterium houstonense*	4.76	G+	Anaerobic
*Porphyromonas endodontalis*	4.56	G-	Anaerobic
*Prevotella buccae*	4.52	G-	Anaerobic
*Prevotella intermedia*	4.03	G-	Anaerobic
*Bacteroides heparinolyticus*	3.12	G-	Anaerobic

### Alpha Diversity Analysis Results

**Figure 2:**
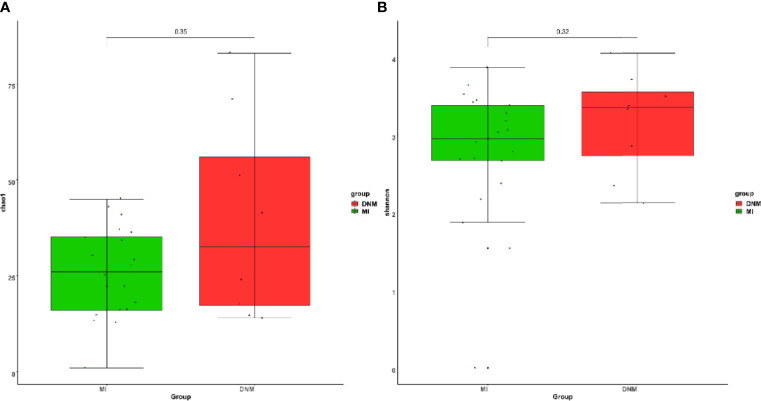
Alpha diversity analysis. **(A)** chao1 index, **(B)** Shannon index.

The Chao1 index is used to estimate the number of species contained in the samples, and a larger Chao1 index means more species in the sample microbiota, i.e., higher richness. The Shannon index was used to estimate the species diversity in the samples, and a larger Shannon value indicates higher microbiota diversity. We found that the species richness of the DNM group was higher than that of the MI group (**Figure 2A**) and that the species diversity of the DNM group was higher than that of the MI group (**Figure 2B**). Differences between the two groups were tested using the Wilcoxon non-parametric test, and the results showed that the differences between the groups were not statistically significant (P > 0.05).

### LEfSe Analysis Results

**Figure 3:**
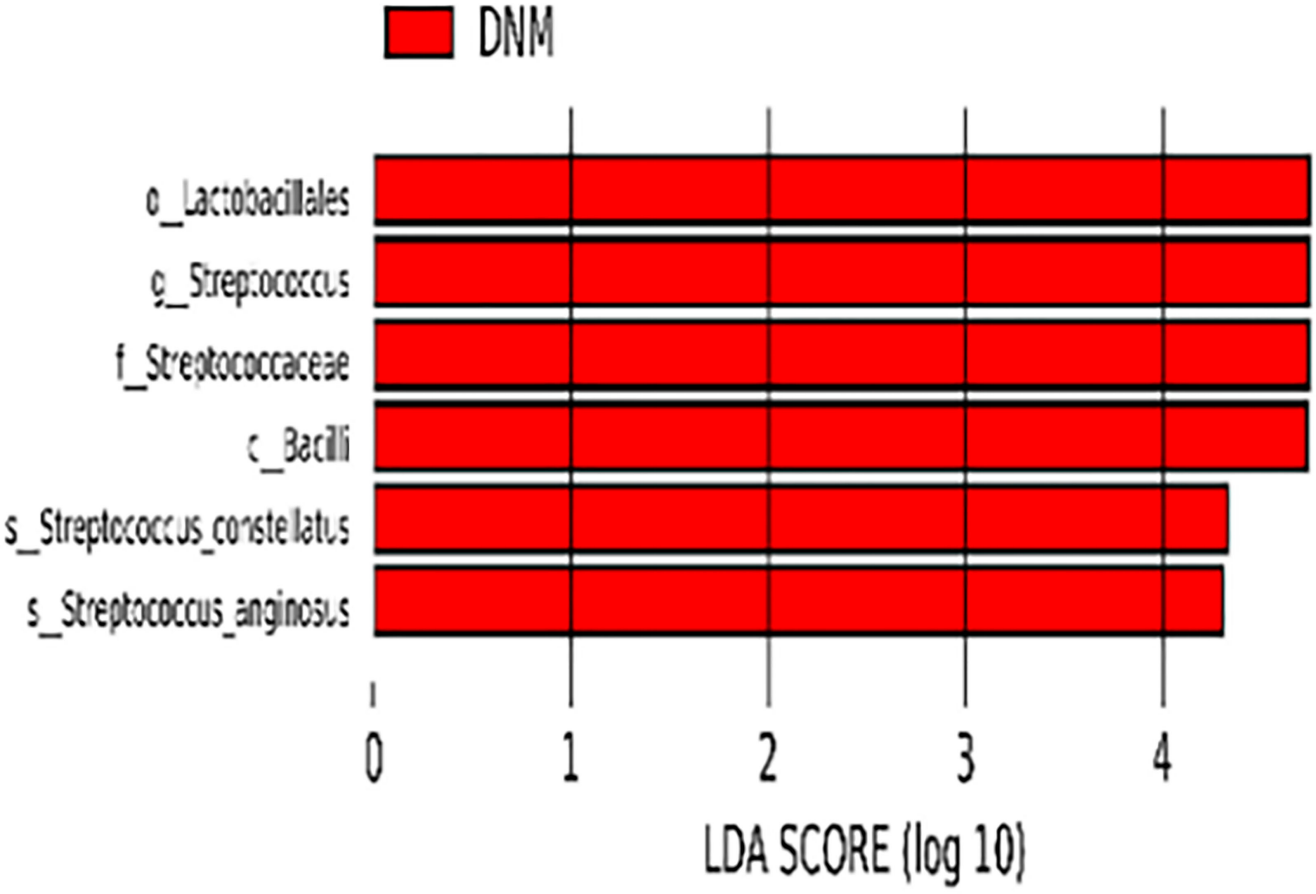
LefSE results.

The species with statistically significant differences between the two groups were further analyzed by LEfSe (P < 0.05) and species that reached the LDAscore threshold (LDA > 4) were: Bacilli, Lactobacilli, Streptococcaceae, *Streptococcus*, *S. constellatus*, and *S. anginosus* (**Figure 3**). These differential bacteria were enriched in the DNM group.

### sPLS-DA Analysis Results

**Figure 4:**
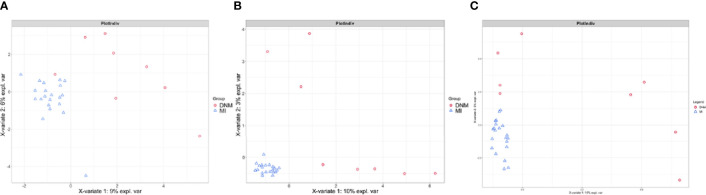
sPLS-DA model for comparison of two groups. **(A)** family level, **(B)** genus level, **(C)** species level.

sPLS-DA is a supervised discriminant analysis statistical method that uses sPLS-DA to model the relationship between the relative abundance of pathogenic bacteria and sample categories to predict sample categories. The sPLS-DA models were developed separately for two-group comparisons (**Figure 4**). Comp1 contributed 9%, 10%, and 10% to the group differences at the family, genus, and species levels, respectively, and comp2 contributed 6%, 3%, and 5% to the group differences at the family, genus, and species levels, respectively. The sPLS-DA model explained the highest degree of comp1 at the species level.

**Figure 5:**
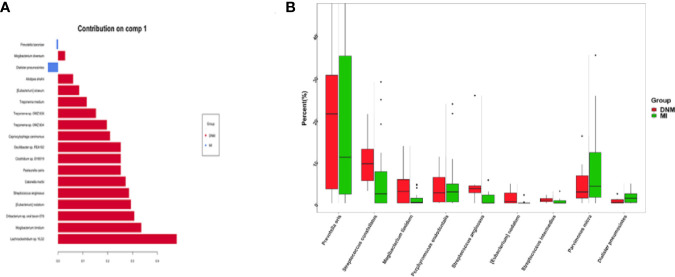
Differential species screened in the sPLS-DA model. **(A)** species-level comp1 axial differential species, **(B)** 9 pathogenic species with high relative abundance in the differential species.

At the species level, the differential species that distinguished the two groups of samples according to the sPLS-DA model were screened on the comp1 axis (**Figure 5A**). Among them, *P. baroniae* and *Dialister pneumosintes* were the dominant bacteria in the MI group, while *Mogibacterium diversum*, *Alistipes shahii*, *Eubacterium siraeum*, *Treponema medium*, *Treponema* sp. *OMZ838*, *Treponema* sp. *OMZ804*, *Capnocytophaga canimorsus*, *Oscillibacter* sp. *PEA192*, *Clostridium* sp. *SY8519*, *Pasteurella canis*, *Catonella morbi*, *S. anginosus*, *S. anginosus*, *E. nodatum*, *Oribacterium* sp. *oral taxon 078*, *M. timidum*, and *Lachnoclostridium.* sp. *YL32* were the dominant organisms in the DNM group. The top nine differential species ranked in relative abundance were plotted in percentage relative abundance (**Figure 5B**). *S. constellatus* (10.14%, 5.67%), *S. anginosus* (5.62%, 1.08%), *S. intermedius* (0.76%, 0.35%), *P. oris* (21.04%,18.59%), *M. timidum* (4.23%, 1.00%), and *E. nodatum* (0.01%, 0.00%) were dominant in the DNM group, while *P. micra* (4.67%, 8.41%), *D. pneumosintes* (0.54%, 1.54%), and *P. endodontalis* (3.89%, 4.56%) were dominant in the MI group.

### Clinical and Microbial Microbiota Correlation

**Figure 6:**
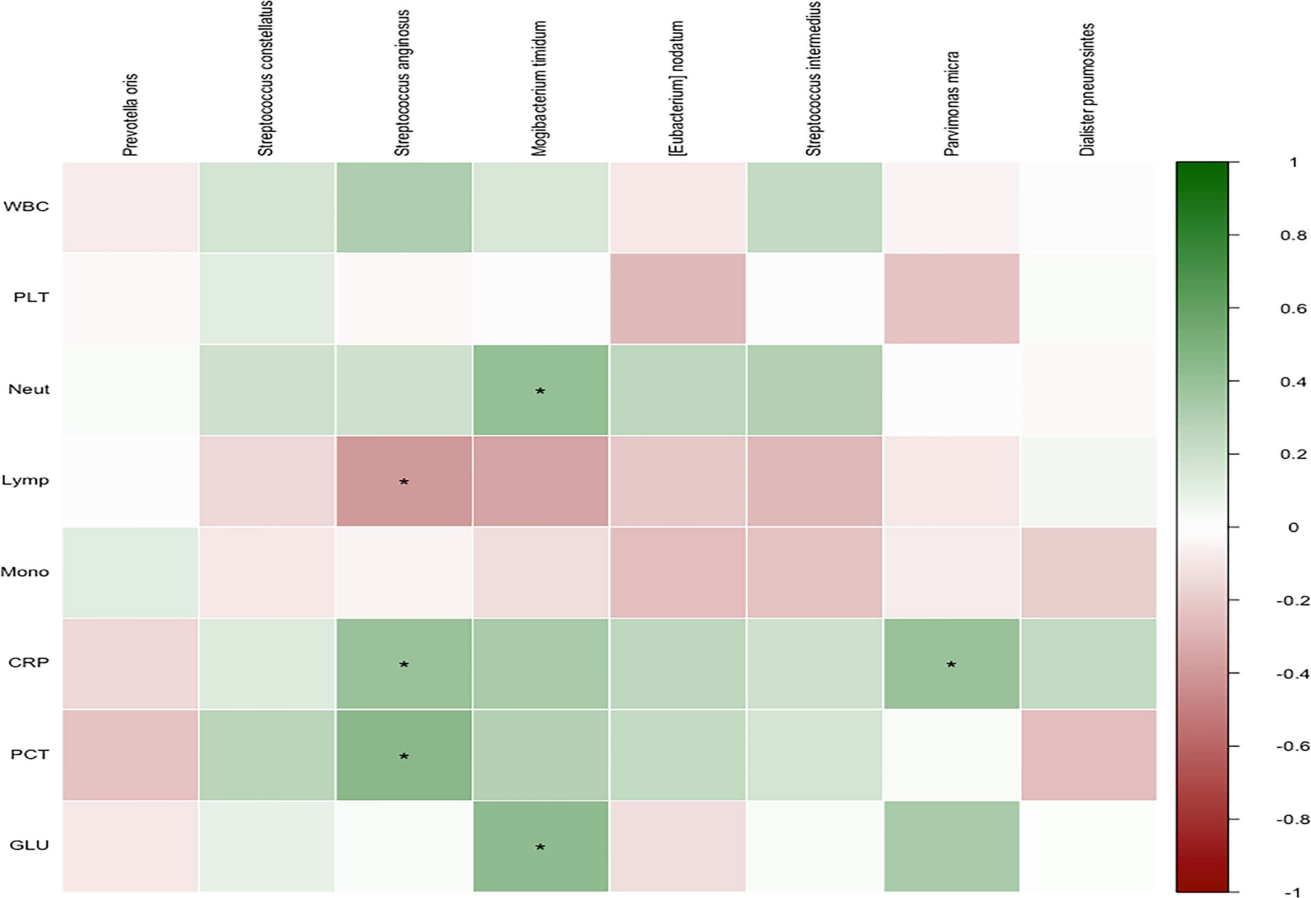
Correlation of members of selected species with covariates. Spearman’s rho was calculated between selected species’ relative abundance and phenotypic scores. Black stars within heatmap boxes indicate significant results (*p < 0.05, corr.test function in psych package of R).

Finally, the clinical characteristics of the patients and the relative abundance of the significant pathogens found in the LefSe and PLS-DA analyses were analyzed (**Figure 6**). Spearman’s rho calculations were used (Bowerman et al., 2020), and black stars in the heat map box indicate significant results (*p < 0.05). The relative abundance of *S. anginosus* was positively correlated with CRP and PCT and negatively correlated with the percentage of lymphocytes. In addition, *M. timidum* was positively correlated with the percentage of neutrophils and glycated hemoglobin, and *P. micra* was positively correlated with CRP.

## Discussion

Bacteria are one of the most common pathogens causing infectious diseases. *In vitro* culture is the standard method for bacterial isolation, but it is generally time-consuming, and many bacteria are not easily cultured, resulting in multiple infections being easily overlooked. The use of mNGS can avoid the limitations of the traditional culture method and directly sequence the sample to rapidly identify known pathogens and effectively detect unknown pathogens. It has been reported that a large proportion of DNM patients present mixed aerobic and anaerobic bacteria in the culture samples (Wheatley M et al., 1990). Frequently detected aerobic pathogens are *Streptococcus*, *Enterococcus*, *Klebsiella*, and *Staphylococcus aureus*, and the most common anaerobes are *Streptococcus pepticus*, *Streptococcus pyogenes*, and *Prevotella (* Ridder et al., 2010; Palma et al., 2015). In the present study, *P. oris*, a non-pigmented, specialized anaerobic bacterium that often causes infections in abscesses, wounds, and soft tissues, was highly abundant in both samples (Goyal et al., 2012; Cahalan et al., 2013). *P. oris* can resist antibiotic treatments and produce more extensive infections in previously infected areas by binding or attaching to cells other than epithelial cells (Shah and Collins, 1990). Studies of *Prevotella* infections in children found that 12% of the infections originated from *P. oris*, 97% of which were mixed infections. Additionally, 38% of the *Prevotella* strains were positive for β-lactamase tests, suggesting that the genus is resistant to β-lactam antibiotics (Brook, 1995; Montagner et al., 2014). A study on the resistance of 192 *Prevotella* strains found that 43.2%, 10.9%, and 19.1% of them were resistant to penicillin, clindamycin, and tetracycline, respectively (Boyanova et al., 2010). Another research team performed drug susceptibility tests on 141 *Prevotella* strains and showed that 49% were non-susceptible to moxifloxacin, with 90% of the *P. oris* strains being non-susceptible (Papaparaskevas et al., 2008). Thus, the selection of antimicrobial drugs should be based on the results of bacterial culture identification and the results of standardized drug susceptibility tests for selective drug administration. However, culture and drug susceptibility testing of anaerobic bacteria require specific conditions and time, and therefore empirical drug use by clinicians is more common.

*S. anginosus* was the dominant organism found in the DNM group, and its relative abundance was increased in the DNM group compared to the MI group. One study reported the progression of the disease in two patients with periodontitis, suggesting that *S. anginosus* group (SAG) oral infections can travel down to the neck and mediastinum and cause thoracic infections (Alegbeleye Bamidele, 2018). Another report of *S. anginosus* infection with a sore throat as the first symptom complicating mediastinal abscess was reported (Jia and Zhao, 2013). In patients with SAG thoracic infection, penicillins are the preferential treatment choice, followed by first- and second-generation cephalosporins, levofloxacin, vancomycin, and linezolid (Wang et al., 2021).

We should also not ignore taxa that are low in abundance but significantly increased in the DNM group. These taxa could be considered as key “microorganisms” that may be more virulent and therefore play a more significant role in the development of an infection. For instance, *M. timidum* has been described as an oral pathogen, usually detected in the subgingival environment and related to the severity of periodontal breakdown (Holdeman et al., 1980). Additionally, the detection of *M. timidum* increased as the severity of the clinical parameters of gingivitis increased (Wittmann et al., 1982), suggesting that this species could contribute to the increased susceptibility of adults to gingivitis and periodontitis. Subsequently, Moore et al. demonstrated the presence of *M. timidum* periodontal pockets of “juvenile” and chronic periodontitis (CP) (Moore et al., 1985). Moreover, *M. timidum* was also correlated to other forms of head and neck infections. Using culture and biochemical techniques, Hill et al. evaluated the microbial biodiversity of head and neck abscesses (Ludwig’s angina) and acute lung and liver infections and identified *M. timidum* in many of the infected sites (Hill et al., 1987). Renato et al. (Casarin et al., 2012) found that diabetic individuals with poor glycemic control presented the highest frequency of *M. timidum* detection.

Recent studies have suggested that the expression level of some inflammatory markers, such as PCT, CRP, WBC, and neutrophil percentage, can correlate with bacterial infections in patients and be used for the early identification of bacterial infections (Chen et al., 2014; Huang et al., 2014; Leli et al., 2015). PCT levels can distinguish Gram-negative bacteria from Gram-positive bacteria and fungi to some extent, aiding clinical diagnosis and treatment (Lorrot et al., 2000). In this study, the relative abundance of *S. anginosus* was positively correlated with CRP and PCT, and negatively correlated with lymphocyte percentage. In addition, *M. timidum* was positively correlated with the percentage of neutrophils and glycated hemoglobin, and *P. micra* was positively correlated with CRP. CRP is an acute-phase protein (Worthmann et al., 2015), one of the non-specific indicators of the acute inflammatory response phase, and is ideal for the early diagnosis of serious bacterial infections. The study of PCT in the identification of the type of pathogenic bacteria in oral and maxillofacial space infections found that Gram-positive bacterial infections can be considered first for patients with detection of PCT > 0.5 mg/L (Hayslip et al., 2009). After the organism is infected with different pathogenic bacteria, the CRP concentration increases significantly earlier than leukocyte indicators and is higher than in non-infectious diseases (Mcwilliam and Riordan, 2010). Therefore, CRP can reflect the bloodstream infection, but has low specificity for identifying different types of pathogenic bacteria (Wacker et al., 2013). WBC, Neut%, and Lymph% are the classical and most common clinical indicators of inflammation and are often used to help diagnose the presence or absence of infection and in the differential diagnosis of bacterial and viral infections (Zhen-Yu et al., 2010; Brodská et al., 2012).

Antibiotics are often used clinically to control infection state, reduce the inflammatory response, and relieve symptoms, but the use of broad-spectrum antibacterial drugs is somewhat blind. The pathogenic bacteria culture analysis of patients with oral and maxillofacial interstitial infections can clarify the direction of antimicrobial drug application. Targeted antibiotic application in the early stage can avoid further aggravation of maxillofacial interstitial infection. Patients with DNM have complex conditions, severe infection, rapid development, and high mortality. The maxillofacial interstitial infection-causing downstream necrotizing mediastinitis has its specific bacteria and characteristic flora changes, and early identification of infecting bacteria can help in effective treatment and in early warning of poor prognosis.

## Data Availability Statement

The data that support the findings of this study have been deposited into CNGB Sequence Archive (CNSA) of China National GeneBank DataBase (CNGBdb) with accession number CNP0003029 https://db.cngb.org/mycngbdb/submissions/project.

## Ethics Statement

The First Affiliated Hospital ethically approved this study of Zhengzhou University (approval number: KY-2019-LW007). The patients/participants provided their written informed consent to participate in this study.

## Author Contributions

ZL and QS conceived and designed the experiments. ZL, PW, ZJ, MS, and SC performed the experiments. PW and ZL analyzed the data. ZL wrote the paper and edited the manuscript. The final manuscript was read and approved by all authors.

## Funding

General Project of Natural Science Fund of Henan Province (212300410391). Joint Construction Project of Henan Provincial Medical Science and Technology Research Plan (LHGJ20200270). Henan Province Medical Science and Technology Research (Key Project jointly built by Provincial Science and Ministry) (SBGJ202002071). Henan Province Young and Middle-aged Health Science and Technology Innovation Talents (Jieqing) Project (YXKC2020030). Special R & D and Promotion of Henan Provincial Department of Science and Technology (212102310592). Medical Appropriate Technology promotion project of Henan Province (SYJS2022112).

## Conflict of Interest

The authors declare that the research was conducted in the absence of any commercial or financial relationships that could be construed as a potential conflict of interest.

## Publisher’s Note

All claims expressed in this article are solely those of the authors and do not necessarily represent those of their affiliated organizations, or those of the publisher, the editors and the reviewers. Any product that may be evaluated in this article, or claim that may be made by its manufacturer, is not guaranteed or endorsed by the publisher.
